# An anatomico-morphometric analysis of proximal femur

**DOI:** 10.6026/973206300200990

**Published:** 2024-09-30

**Authors:** Gaurangi Bhimsaria, Nagaeswari T., Srimathi T., Kalpana Ramachandran

**Affiliations:** 1 Department of Anatomy, Sri Ramachandra Medical College and Research Institute, SRIHER (DU), Porur, Chennai 600 116, Tamil Nadu, India

**Keywords:** Anthropometry, femur, femoral head, hip joint, hip prosthesis, morphometry

## Abstract

The proximal femur is an important area of interest as it is involved in the articulation of the hip joint and it contributes to the mechanics of locomotion and the weight bearing capacity of the femur. Pathologies like osteoporosis and fractures are common in this site, and surgical interventions like hip arthroplasty and internal fixtures with implants rely on knowledge about the anatomy of the proximal femur to ensure effective treatment. Hence, a greater understanding of these variations will be helpful in constructing better fitted prosthesis. In this study, six morphometric parameters of the proximal femur were studied and measured in 100 dry adult femur bones - head diameter, transverse diameter of fovea, longitudinal diameter of fovea, diameter of neck, intertrochanteric line length, neck shaft angle and correlations were noted. The data obtained is specific for the southern Indian population and hopes to aid in production of better fitted hip prostheses for patients.

## Background:

In the USA alone 5,44,000 hip arthroplasties are done on an average each year and in India the number is set to reach an all-time high by the year 2026. By the year 2050 the total number of hip fractures is projected to be double the number of cases confirmed in 2018. [1] With the rise in the number of surgeries involving the hip joint, a detailed analysis of the proximal femur and measurements of its parts will aid in creating finer adjusted and better suited prostheses in addition to implants which will in turn help in improving treatment outcomes. Various studies conducted in contemporary times have shown that these parameters of the femur are affected by a person's ethnicity, gender, lifestyle and environmental factors. Studies conducted in various parts of India have noted a difference in values due to influence of such factors [2, 3]. Therefore, it is of interest to observe the proximal end of femur and to report variations present proximal end of femurs of southern Indian population along with other correlations.

## Methodology:

The materials used are Vernier Caliper, Goniometer and Dry femur bones. 100 dry adult femur bones are taken from Department of Anatomy in Sri Ramachandra Medical College. for the study, nature of which is observational and descriptive. Adult femurs which are intact, dried and non-pathological were included and femurs which have any arthritic deformity or gross damage were excluded in this study. Bones of the right and left side will be separated. The parameters measured are the diameter of head of femur (the linear distance measured between the upper and lower end of a femoral head in the cranio-caudal axis), transverse diameter of fovea(fullest extent of fovea capitis along the transverse axis), longitudinal diameter of fovea (fullest extent of fovea capitis along vertical axis), diameter of neck (the linear distance measured between the upper and lower end of the anatomical neck in the cranio-caudal axis), length of the intertrochanteric line (the total extent lengthwise of the intertrochanteric line) and the neck shaft angle with help of a vernier calliper with a least count of 0.01mm and goniometer with a least count of 1 degree. The values obtained will be recorded in Microsoft Excel. Then the values will be rounded up to two decimals and the mean along with standard deviation will be calculated. The data was tabulated and its statistical analysis was done using SPSS software. The findings of the six parameters measured are recorded in Table 2.

## Results:

The head diameter of the femur was found to be 38.16±3.00 mm. The transverse diameter of the fovea was found to be 10.97±2.20 mm whereas the longitudinal diameter was found to be 9.46±2.24 mm. The neck diameter was found to be 28.62±3.33 mm. Intertrochanteric length was 54.14±5.91 mm. The angle between neck and shaft was found to be 133.89°±5.95° (Table 1). A positive correlation was noted between head diameter and longitudinal diameter of fovea (Table 3). A significant positive correlation was also noted between the head diameter and neck diameter (Table 4).

## Discussion:

Various methods have been used to measure the bony proximal femur's mentioned parameters-cadaveric morphometry, computed tomography, ultrasound and magnetic resonance imaging. The results obtained in this study are similar to other studies conducted in the Indian population (Table 5). Studies done in different population's revealed remarkable differences in these indices, showing that there is a need to customize prosthetic design that caters to every individual [2]. The fovea capitis is also an important anatomical structure in the proximal femur that transmits vessels supplying the femoral head. This ligament plays a role in cases where the head of femur undergoes avascular necrosis, which is a complication of hip fractures and dislocations. The mean transverse diameter of the fovea in our study was 10.97±2.20 mm. This value is comparable to those found by Gupta *et al.*, i.e. 11.38±2.35 mm. However, the longitudinal diameter of the fovea in our study, 9.46±2.24, is much lesser than the value obtained by the same study 15.94±3.37 mm, suggesting a regional variation between northern and southern Indian populations. The computed tomography study done by Ceynowa *et al.* [5] in Poland found the transverse diameter to be 12.94±2.61 mm and the longitudinal diameter to be 10.83±2.32 mm, with the values being greater in men than in women. The intertrochanteric length was found to be 54.14±5.91 mm which is lesser than the value of 60.31±7.33 mm obtained in the dry bone study by Resmi George & Nithin K Raju [6]. The mean neck shaft angle obtained in this study is larger than the values obtained by Isaac B. *et al.* [7] and Late SV & Keche H, [8] but were similar to the values obtained by Kamath SU *et al.* [9] and Haddad B *et al.* [10]

Interpretation of the values obtained in this study exhibit significant positive correlations between the longitudinal diameter of the fovea and the head diameter. Maximum positive correlation was seen between the neck diameter and the vertical head diameter.

## Conclusion:

The morphometric analysis performed on the proximal femur provides valuable insights into anatomical variations and structural characteristics of the bone. The proximal femoral morphometry has notable differences among different groups of populations and such differences must be carefully analysed and taken into consideration while designing prostheses and in surgical interventions. The results obtained through this study will add to the existing database that will be helpful in designing implants, plates and prostheses for hip reconstructive surgeries that are catered to the Southern Indian population.

## Figures and Tables

**Table 1:** Statistical summary of the proximal femur's morphometry

**Parameter**	**N**	**Mean**	**Std Dev**	**Minimum**	**Maximum**
Head Diameter	100	38.16	3	30	43
Transverse Diameter of Fovea	100	10.97	2.2	4	21
Longitudinal Diameter of Fovea	100	9.46	2.24	4	16
Diameter of Neck	100	28.62	3.33	21	36
Intertrochanteric Length	100	54.14	5.91	39	70
Neck-Shaft Angle	100	133.9	5.95	122	153

**Table 2:** Morphometric variables of right and left side femurs

**Parameter**	**Group**	**N**	**Mean**	**Standard Deviation**	**S.E. Mean**
Head Diameter	L	50	38.32	2.75	0.39
	R	50	38	3.25	0.46
Transverse Diameter of Fovea	L	50	11.61	1.92	0.27
	R	50	10.34	2.29	0.32
Longitudinal Diameter of Fovea	L	50	9.85	2.02	0.29
	R	50	9.07	2.39	0.34
Diameter of Neck	L	50	28.95	3.14	0.44
	R	50	28.29	3.51	0.5
Intertrochanteric Length	L	50	52.09	5.11	0.72
	R	50	56.19	6	0.85
Neck-Shaft Angle	L	50	132.4	5.36	0.76
	R	50	135.38	6.19	0.88

**Table 3:** Correlation between head diameter of a femur and longitudinal diameter of fovea

		**Head Diameter**	**Longitudinal Diameter of Fovea**
Head Diameter	Pearson Correlation	1	0.511
	Sig. (2-tailed)		0
	N	100	100
Longitudinal	Pearson Correlation	0.511	1
Diameter of Fovea	Sig. (2-tailed)	0	
	N	100	100

**Table 4:** Correlation between head diameter and neck diameter of proximal femur

		**Head Diameter**	**Neck Diameter**
Head Diameter	Pearson Correlation	1	0.735
	Sig. (2-tailed)		0
	N	100	100
Neck Diameter	Pearson Correlation	0.735	1
	Sig. (2-tailed)	0	
	N	100	100

**Table 5:** Comparison between different studies of proximal femur morphometry in Indian population

**Parameter**	**Verma *et al.*[2]**	**Gupta *et al.*[3]**	**Lingamdenne PE *et al.* [4]**	**Present Study**
Head Diameter (in mm)	42.32±4.11	41.59±3.25	42.3±5.40	38.16±3.00
Neck Diameter (in mm)	33.02±4.22	29.45±3.33	24.8±2.30	28.62±3.33
Neck Shaft Angle (in degrees)	128.90°±4.49°	119.08°±5.18°	119.44°±4.13°	133.89°±5.95°
